# Genetically Modified α-Amylase Inhibitor Peas Are Not Specifically Allergenic in Mice

**DOI:** 10.1371/journal.pone.0052972

**Published:** 2013-01-09

**Authors:** Rui-Yun Lee, Daniela Reiner, Gerhard Dekan, Andrew E. Moore, T. J. V. Higgins, Michelle M. Epstein

**Affiliations:** 1 Division of Immunology, Allergy and Infectious Diseases, Experimental Allergy, Department of Dermatology, Medical University of Vienna, Vienna, Austria; 2 Institute of Clinical Pathology, Medical University of Vienna, Vienna, Austria; 3 CSIRO Plant Industry, Canberra, ACT, Australia; Université Libre de Bruxelles, Belgium

## Abstract

Weevils can devastate food legumes in developing countries, but genetically modified peas (*Pisum sativum*), chickpeas and cowpeas expressing the gene for alpha-amylase inhibitor-1 (αAI) from the common bean (*Phaseolus vulgaris*) are completely protected from weevil destruction. αAI is seed-specific, accumulated at high levels and undergoes post-translational modification as it traverses the seed endomembrane system. This modification was thought to be responsible for the reported allergenicity in mice of the transgenic pea but not the bean. Here, we observed that transgenic αAI peas, chickpeas and cowpeas as well as non-transgenic beans were all allergenic in BALB/c mice. Even consuming non-transgenic peas lacking αAI led to an anti-αAI response due to a cross-reactive response to pea lectin. Our data demonstrate that αAI transgenic peas are not more allergenic than beans or non-transgenic peas in mice. This study illustrates the importance of repeat experiments in independent laboratories and the potential for unexpected cross-reactive allergic responses upon consumption of plant products in mice.

## Introduction

Genetically modified (GM) crop areas have increased rapidly since their introduction in 1996 [1]. New approaches to generate plants that are resistant to insect infestation are being actively sought, especially to reduce reliance on chemical insecticides. For example, genetically modified peas (*Pisum sativum*), chickpeas (*Cicer arietinum*) and cowpeas (*Vigna unguiculata*) expressing the gene for alpha-amylase inhibitor-1 (αAI) from the common bean (*Phaseolus vulgaris*) cultivar Tendergreen are completely protected from weevil destruction [2], [3], [4]. αAI is seed-specific, accumulated at high levels and undergoes post-translational modification as it traverses the seed endomembrane system [5]. The excellent insecticidal effect of αAI [6] and the long-term safe consumption of beans containing αAI [7] make it a promising gene to insert into insect-susceptible legumes. However, one study suggested that αAI peas expressed a variant protein resulting in allergic responses in mice to the peas but not the beans [8]. They found that mice consuming αAI peas developed elevated levels of αAI-specific IgG1 but not IgE antibodies, had enhanced delayed-type hypersensitivity responses and increased reactivity to other allergens (adjuvant effect) whereas mice fed non-transgenic peas and Pinto beans had no αAI reaction. Mass spectrometry results revealed differences in post-translational modifications, which the authors suggested led to the reported allergenicity. These results were received with some skepticism including an editorial in Nature Biotechnology [9].

More recently, a comparison using high-resolution mass spectrometry of αAI from bean and transgenic legume sources revealed heterogeneous structural variations in peas and beans due to differences in glycan and carboxypeptidase processing, but the transgenic versions were within the range of those observed from several bean varieties [5]. Moreover, when purified αAIs from beans and transgenic peas were used to immunize mice, all elicited Th1 and Th2- type αAI-specific antibodies [5]. This questions the reported enhanced αAI transgenic pea-specific immunogenicity and allergenicity compared with the naturally occurring protein in beans.

The objective of this study was to evaluate allergenicity of αAI peas, cowpeas and chickpeas and compare them to non-transgenic controls, Pinto and Tendergreen beans (the latter was the source of αAI gene) in mice. To achieve this aim, we evaluated the immunogenicity and allergenicity of αAIs from these transgenic legumes to determine whether the transgenic αAIs were more allergenic than the αAIs from Pinto and Tendergreen beans. The evaluation included a comparison of antibody titres to αAIs from each source. Additionally, we tested the antibody response to twice weekly consumption of the pea, cowpea, chickpea and bean meals for 4 weeks. After the feeding period, we challenged the respiratory tract with αAI to evaluate *in vivo* T lymphocyte responses. Lastly, we assessed the adjuvant effect of αAI pea consumption on the initiation and exacerbation of non-cross-reactive ovalbumin (OVA)-induced allergic lung disease.

## Materials and Methods

### Ethics Statement

This study was carried out in strict accordance with the guidelines for the care and use of laboratory animals of the Austrian Ministry of Science. The protocol was approved by the Committee on the Ethics of the Austrian Ministry of Science (Number: GZ: 68.205/0237-II/3b/2010). All painful procedures were performed under anesthesia, and all efforts were made to minimize suffering.

### Mice

Female BALB/c mice (6–8 week old) were purchased from Charles River (Germany). Mice were provided with tap water ad libitum throughout the study and were maintained in the University of Veterinary Medicine animal facility in Vienna, Austria. We accommodated 8 mice per Type III cage with stainless steel covers using a 12 h light/dark schedule, at temperature of approximately 22°C. Mice were observed two times daily. The basal diet was OVA-free autoclaved SSNIFF V1126-000, from Soest, Germany: (http://www.ssniff.de/documents/03_katalog_dt_maus_ratte.pdf) provided *ad libitum*. All experiments used 8 animals per group.

### Isolation of α-Amylase Inhibitors

The transformation of peas, chickpeas and cowpeas for seed-specific expression of the αAI gene from the common bean (*P. vulgaris*, cv Tendergreen) has been described previously [2], [3], [10]. Seed meals from the transgenic legumes, Pinto and Tendergreen beans have approximately the same concentration of αAI and are in the range 2–4% of total seed protein [5]. αAIs from the seeds of the various beans and transgenic legumes were purified as previously described [11]. Briefly, seed meals from Pinto bean, Tendergreen bean, and transgenic peas, cowpeas and chickpeas were extracted with a NaCl solution (1%) followed by a heat treatment (70°C), dialysis and centrifugation. The inhibitors were enriched by anion exchange (DEAE-Sepharose CL-6B, Pharmacia) and gel filtration (Sephacryl S-200, Pharmacia) chromatography. Active fractions were determined by inhibition of porcine pancreatic α-amylase (Ceralpha: α-Amylase Assay Kit, Megazyme International, Ireland) and the most pure fractions were determined by inspection of Coomassie-stained 15–25% SDS-PAGE gels. Finally, the appropriate pooled fractions were dialysed against water, lyophilized and stored at 4°C. The proteins were highly purified as can be assessed from the mass spectrometric analyses described earlier [5]. Pea lectin was purified as described earlier [12]. The level of pea lectin in peas [13] is comparable to the level of αAI in the peas [5]. Pea lectin is structurally related to αAI [14] and their amino acid sequences are 38% identical and 54% similar to each other as determined by Blast® analysis. Purified proteins contained low or undetectable levels of endotoxin (Andrew Moore, unpublished data).

### αAI feeding and immunization protocols

*Intraperitoneal immunization:* Naïve mice received i.p. injections of 10 µg of purified αAIs from either αAI pea, Tendergreen bean, Pinto bean, or pea lectin in 200 µl PBS on days 0 and 21. One week later, sera were taken and stored at −20°C until use in ELISAs measuring anti-αAI or pea lectin-specific antibody titres. *Intranasal immunization:* In separate experiments, we instilled naïve mice with 50 µg of purified αAI dissolved in 50 µl PBS into the nares, so that it reaches the lungs, on days 0, 2, 4, 14, 16, 18 and tested for anti-αAI-specific antibody titres and allergic lung inflammation and mucus production on day 21. *Pea and bean feeding for the evaluation of allergic responses to* αAI: Feeding experiments were done by gavage (intragastric administration). Mice were gavaged suspensions of 100 mg/ml in 250 µl of PBS raw or 100°C heat-treated seed meals of αAI -pea, -cowpea, -chickpea and non-transgenic pea, Pinto bean and Tendergreen bean twice weekly for 4 consecutive weeks using the same protocol as in Prescott et al. [8]. As a read out of allergic sensitization during feeding, at 96 h after the final gavage, mice received one intranasal instillation of 50 µg of αAI purified from αAI pea or Tendergreen bean dissolved in 50 µl of PBS as a lung challenge. The mice were then evaluated 72 h later for antibody titres, allergic lung inflammation and mucus production. *Adjuvant studies:* Mice were gavaged suspensions of 100 mg raw seed meals of αAI pea, non-transgenic pea, Pinto bean and Tendergreen bean in 250 µl of PBS twice weekly for 4 consecutive weeks, 1 month before the initiation and exacerbation of OVA-induced allergic asthma (see protocol below). Both heat-treated and raw seed meals were used in these studies to determine whether there were differences between seed meals with denatured proteins.

### Induction of OVA-induced allergic asthma

Mice were immunized with 10 µg of OVA (Sigma Chemical Co., St. Louis, MO) i.p. on days 0 and 21. Mice were challenged 1 week later with nebulized 1% OVA in PBS in a Plexiglas chamber by an ultrasonic nebulizer (Aerodyne, Kendall, Neustadt, Germany) for 60 min twice daily on days 28, 29 for disease initiation. For disease exacerbation, mice were allowed to recuperate from acute disease and were then nebulized on days 91 and 92. Three days after the last aerosol challenges, the mice were evaluated for antibody titres, allergic lung inflammation and mucus production.

### Lung inflammation and mucus hypersecretion

*Airway inflammation:* Mice were terminally anesthetized 72 h after the last antigen challenge. The mice were then subjected to tracheotomy followed by the lavage of the lungs 3 times with PBS for a total volume of 1 ml to collect bronchoalveolar lavage fluid (BAL). The total number of cells in BAL was enumerated (Neubauer hemocytometer) and the differential cell counts were determined by morphological examination of at least 300 cells in cytocentrifuged preparations (Cytospin-4, Shandon Instruments, UK), stained with Kwik-Diff (Thermo Fisher Scientific Inc., Pittsburgh, PA).

After BAL, lungs were fixed by immersion in 4% paraformaldehyde and then embedded in paraplast. Lung sections of 3 µm were stained with hematoxylin and eosin (H&E) for morphological evaluation, with Luna stain for eosinophil enumeration and with Periodic-acid-Schiff reagent (PAS) for detection of mucus within the lung epithelium. For scoring of inflammatory cell infiltration, sections containing main stem bronchi from each lung specimen stained with H&E were used. Blinded observers graded the extent of inflammation in the lungs according to a semi-quantitative scoring system: Grade 0: no inflammatory infiltrates; Grade 1: inflammatory infiltrates in central airways; Grade 2: inflammatory infiltrates extending to middle third of lung parenchyma; and Grade 3: inflammatory infiltrates extending to periphery of the lung. We enumerated eosinophil counts in lung sections stained with Luna by counting ten random fields (40× magnification) containing alveoli but without major airways or vessels on low power magnification, and averaged the counts for each lung section. For detection of mucus-secreting cells, adjacent lung sections were stained with PAS and counter stained with hematoxylin. We used the following scoring system for mucus production: Grade 0 – no mucus producing cells in airways; Grade 1: 0–20%; Grade 2: 21–40%; Grade 3: 41–60%; Grade 4: 61–80 and Grade 5: 81–100% mucus producing cells in airway walls stained for mucopolysaccharide.

### Serum OVA- and αAI-specific immunoglobulin

For the measurement of antigen-specific immunoglobulin IgG1, IgG2a and IgE, ELISA plates were coated with OVA, purified αAI or pea lectin at 10 µg/ml overnight at 4°C. The plates were then washed and blocked with 2% bovine serum albumin in PBS for 2 h at RT. The plates were washed and sera were added and incubated for 24 h at 4°C. Plates were washed again and then incubated with biotinylated anti-IgG1 for an additional 2 h at 4°C (Southernbiotech, Birmingham, AL), anti-IgE (Becton Dickinson Biosciences, Franklin Lakes, NJ) or anti-IgG2a (Southernbiotech) detection mAbs, followed by incubation with streptavidin horseradish peroxidase (Southernbiotech) for 1 h at RT. Plates were washed and incubated with a TMB substrate solution (100 µl/well, BD OptEIATM, Becton Dickinson Biosciences) for 10 min at RT. The reaction was stopped with 100 µl of 0.18 N H_2_SO_4_ and absorbance was measured at 450 nm.

### Statistical analysis

Groups were compared with the Kruskal-Wallis test followed by the Dunn's multiple comparison test and the Mann Whitney test for grading histology using GraphPad Instat v.5.0 (GraphPad Software Inc.). p values were considered significant at <0.05.

## Results and Discussion

**Figure 1:**
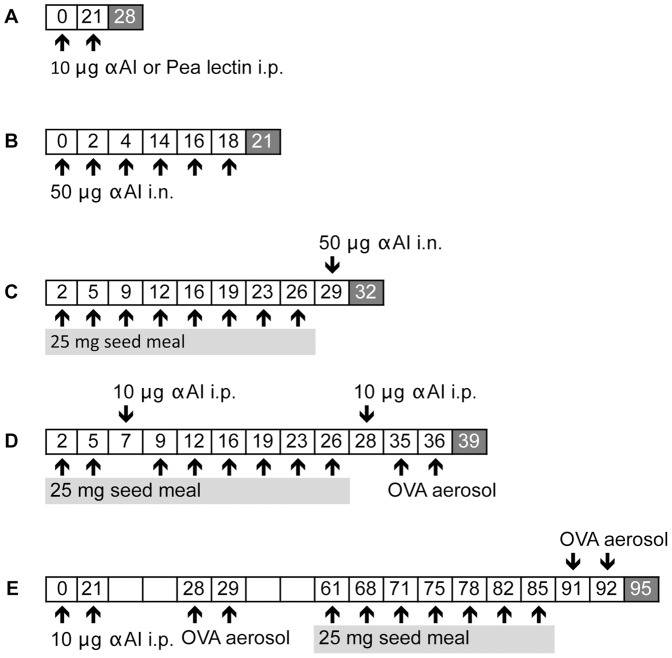
Experimental protocols. A. *Intraperitoneal immunization with purified proteins to assess protein immunogenicity.* On days 0 and 21 mice were immunized with 10 µg of purified αAIs from the transgenic peas, Tendergreen bean, Pinto bean, or pea lectin purified from non-transgenic peas. On day 28, sera were harvested and evaluated for IgG1, IgE and IgGa2 antibodies to αAI. B. *Intranasal immunization with purified proteins to assess differences in protein allergenicity.* On the indicated days, mice were instilled with 50 µg of purified αAIs from transgenic peas, Tendergreen bean, Pinto bean and tested for antibody titres and allergic lung responses on day 21. C. *Seed meal feeding for the evaluation of immune responses to αAI upon ingestion.* Mice were gavaged with 25 mg seed meals from αAI -pea, -cowpea, -chickpea, non-transgenic peas, Pinto bean and Tendergreen bean 8 times on the indicated days. On day 29, mice received an intranasal instillation of 50 µg of αAI purified from αAI pea or Tendergreen bean, and were evaluated on day 32 for antibody titres and allergic lung responses. D. *Adjuvant effect of peas and beans on the initiation of OVA-induced allergic lung disease.* Mice were gavaged with 25 mg raw or cooked seed meals from αAI -pea, -cowpea, -chickpea, non-transgenic peas, Pinto bean and Tendergreen bean 8 times on the indicated days. Mice were immunized to induce allergic disease with 10 µg of OVA on days 7 and 28. After one week, the mice were nebulized with OVA on days 35 and 36. On day 39, antibody titres and allergic lung responses were measured. E. *Adjuvant effect of peas and beans on the exacerbation of OVA-induced allergic lung disease.* Mice were induced with allergic disease on days 0 and 21 and aerosolized on days 28 and 29 and then allowed to recuperate. On the indicated days mice were gavaged 8 times with 25 mg raw or cooked seed meals. One day later, mice were nebulized with OVA on 2 consecutive days to induce a disease exacerbation. On day 90, they were evaluated for antibody titres and allergic lung responses.

**Figure 2:**
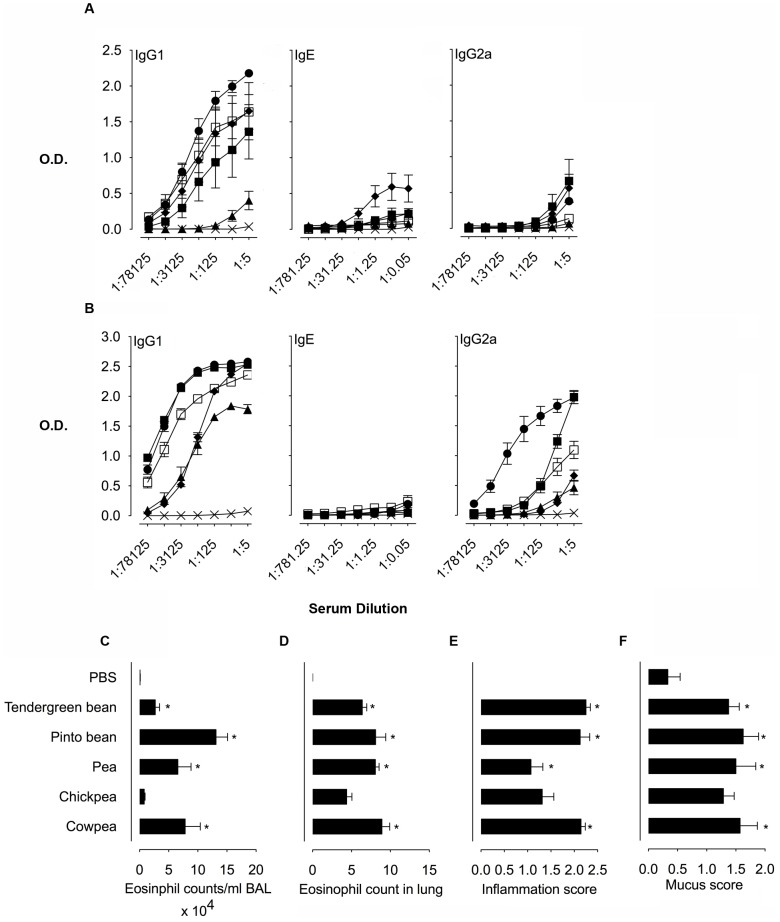
Immune responses to αAIs upon intranasal and intraperitoneal immunization. Serum antibody titres for Anti-αAI IgG1, IgE and IgG2a from A. i.p. αAI immunized mice and B. from i.n. αAI immunized mice. The treatment groups for A and B include PBS only ×, purified αAI proteins from Tendergreen bean ▪, Pinto bean □, pea ▴, chickpea ⧫, and cowpea •. Data are expressed as mean OD_450 nm_ ± SEM; n = 8, duplicate samples. For IgE, dilutions are expressed ×10^3^. C. Eosinophil counts in BAL fluid from mice immunized with i.n. αAIs. D. Eosinophil counts in Luna-stained lung sections from mice immunized with i.n. αAIs. E. Inflammation scores of lung sections from mice immunized with i.n. αAIs. F. Mucus scores in PAS-stained lung sections from mice immunized with i.n. αAIs. Data are expressed as means ± SEM; n = 8. For eosinophil counts in BAL and lungs, data were compared using the Kruskal-Wallis test followed by Dunn's multiple comparison test. For histological scoring, data were compared with the Mann Whitney test. *p<0.05 for all groups above the PBS controls. These are representative data from 2 experiments.

The scheme in Figure 1 illustrates the experimental protocols. We first tested the hypothesis that different post-translational modifications to αAI in pea alters immunogenicity and allergenicity compared to αAI in bean. To directly investigate αAI immunogenicity, we immunized mice with purified αAI from Pinto bean, Tendergreen bean and transgenic pea, cowpea and chickpea by i.p. (Fig. 1A) or i.n. (Fig. 1B) routes. We administered 10 µg of αAI without adjuvant i.p. 3 weeks apart and measured anti-αAI-specific IgG1, IgG2a and IgE serum titres one week later (Fig. 2A). To further assess the *in vivo* allergic response induced by αAI, we immunized mice i.n. with 50 µg of αAI 6 times over a 3-week period and then evaluated antibody titres and lung responses (Fig. 2B).

Intraperitoneal immunization with all αAIs led to increased allergic isotype, anti-αAI-specific IgG1 responses (Fig. 2A) and confirmed previous data [5]. Cowpea, Pinto bean and chickpea αAIs generated the highest IgG1 titres, whereas Tendergreen bean αAI resulted in a slightly lower titre and pea αAI was the least immunogenic. Anti-αAI-specific IgE levels were low for all groups with chickpea αAI having the highest titre. Generally, the allergy IgE antibody isotype responses are 10–100 fold lower than allergic IgG1 isotype in mice (M. Epstein, unpublished results). Because we used protocols intended to skew responses towards allergic Th2 isotypes, IgG2a titres were, as expected, lower than IgG1. Tendergreen bean, chickpea and cowpea αAIs induced the highest IgG2a titres. Although there are distinct patterns of glycosylation of αAIs [5] that may explain the magnitude of antibody responses, there was no apparent correlation between anti-αAI titres and the source of the αAI.

Intranasal αAI administration led to high anti-αAI-specific IgG1 titres against cowpea and Tendergreen bean αAIs, followed by lower titres against Pinto bean and chickpea αAIs and the lowest titres were against pea αAI (Fig. 2B). Anti-αAI IgE responses were low for all αAIs. Interestingly, IgG2a titres were higher for i.n. compared to i.p. αAI administration. This is probably related to the higher total i.n. dose of 300 µg of αAI compared with a total αAI i.p. dose of 20 µg. Thus, both IgG1 and IgG2a isotype titres were higher in i.n. compared to i.p. experiments. Cowpea and Tendergreen bean αAIs induced the highest anti-αAI-specific IgG2a titres followed, in order, by Pinto bean, chickpea and pea. Immunization by i.n. and i.p. routes demonstrated that antibody responses to αAI from beans and transgenic peas differed but the transgenic proteins were not more immunogenic or allergenic than bean αAIs.

Except for chickpea αAI, intranasal administration of all αAIs induced significant airway and lung inflammation when compared to PBS (Fig. 2C–E). Pinto bean and cowpea αAI induced the highest eosinophil infiltration in the airways with approximately 20 and 12% eosinophils within the infiltrates, respectively. αAI from pea, Tendergreen bean and chickpea induced approximately 11, 5 and 3% eosinophils in BAL fluid, respectively (Fig. 2C). Pinto bean αAI-induced airway eosinophilia is statistically greater than eosinophilia induced by Tendergreen bean and chickpea αAIs. Enumeration of eosinophils in lung tissue sections revealed that immunization with all αAIs induced significant allergic inflammation compared to PBS controls (Fig. 2D). Tendergreen and chickpea αAIs appeared to induce more allergic inflammation in lungs, but there were no statistical differences between any of the αAI-immunized groups. Similarly, all αAI-immunized mice developed extensive inflammatory infiltrates in contrast to PBS control sections that had low or no inflammation (Fig. 2E and Fig. S1). Analysis of PAS-stained lung sections revealed that all groups had similar mucus secretion responses to i.n. protein immunization compared to low or no mucus production in PBS controls (Fig. 2F and Fig. S1). Taken together, these data illustrate that when administered as per our protocols, αAI, irrespective of source is immunogenic and allergenic in mice. Variations in immune responses may be related to differential post-translational modifications such as glycosylation as previously reported [5]. However, no correlation could be made between immunogenicity and allergenicity of αAIs from bean and the transgenic legumes.

To evaluate whether consumption of bean and αAI pea seed meals generated allergic responses to αAI, we fed mice αAI transgenic peas, non-transgenic (nGM) peas, Tendergreen bean and Pinto bean (Fig. 1C). Mice received raw or heat-treated seed meal diluted in PBS twice weekly for 4 consecutive weeks, followed by 50 µg of αAI i.n. This intranasal exposure was added as an indication of *in vivo* T lymphocyte activation following ingestion of seed meal containing αAI. We then measured allergic airway and lung inflammation, mucus hypersecretion and antibody production as a readout for an αAI-specific immune response.

**Figure 3:**
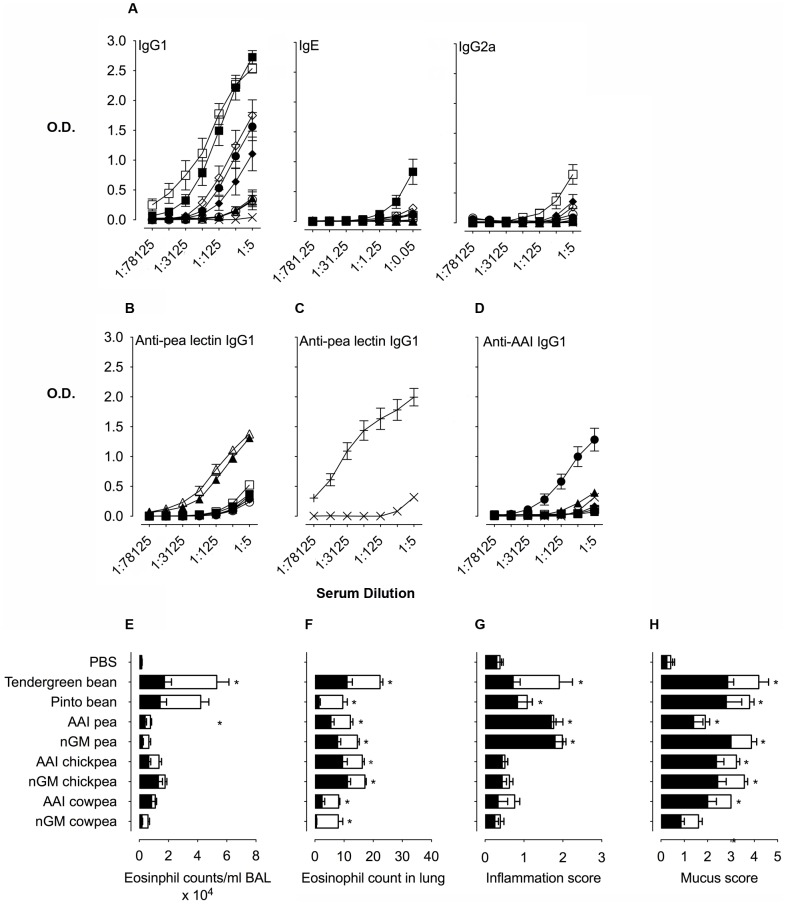
Immune responses following consumption of raw or cooked seed meal from αAI pea and bean. A. Serum antibody titres for anti-αAI IgG1, IgE and IgG2a from mice gavaged PBS or seed meals. B. Serum antibody titres for anti-pea lectin IgG1 from mice gavaged PBS or seed meals. C. Serum antibody titres for anti-pea lectin IgG1 from mice immunized i.p. with either PBS or pea lectin +. D. Serum IgG1 antibody titres of mice immunized with i.p. with pea lectin against αAI proteins purified from pea, cowpea, chickpea, Pinto bean and Tendergreen bean. Groups include PBS alone ×, Tendergreen bean ▪, Pinto bean □, αAI pea ▴, nGM pea ▵, αAI chickpea ⧫, nGM chickpea ◊, αAI cowpea • and nGM cowpea ○. IgE dilutions are expressed ×10^3^. Data are expressed as mean OD_450 nm_ ± SEM; n = 8, duplicate samples. Allergic lung inflammation evaluated by E. Eosinophil counts in BAL fluid, F. Eosinophil counts in Luna-stained lung sections, and G. Inflammation scores of lung sections. H. Allergen-induced mucus production is graded using mucus scores in PAS-stained lung sections. Raw (filled bars), cooked (open bars). Data are expressed as means ± SEM; n = 8. For eosinophil counts in BAL and lungs, data were compared using the Kruskal-Wallis test followed by Dunn's multiple comparison test. For histological scoring, data were compared with the Mann Whitney test. *p<0.05 for all groups above the PBS controls. These are representative data from 2 experiments.

Serum antibody titres tested 72 hours after the i.n. instillation showed that consumption of all raw seed meal suspensions including nGM seed meal plus αAI i.n. exposure led to the production of anti-αAI-specific antibodies (Fig. 3A). Serum titres measured from mice before and after i.n. αAI were similar (data not shown) and naïve mice administered one i.n. dose of αAI did not induce immune responses (data not shown). The titres were highest for Tendergreen bean>Pinto bean>nGM chickpea>αAI cowpea>αAI chickpea>nGM cowpea = αAI pea = nGM pea. Indeed, nGM chickpea serum titres were even higher than the titres in serum from animals fed transgenic seed meals. Anti-αAI IgE and IgG2a titres were lower than that of IgG1 and IgE and IgG2a titres were highest in mice fed bean seed meal.

Due to the antibody response observed upon feeding nGM peas, we sought to identify whether there was a protein in the nGM pea that was crossreactive with αAI. Because of the known homology of pea lectin with αAI, we compared antibody reactivity of pea lectin from nGM peas with bean αAI using separate approaches. Firstly, we measured anti-pea lectin IgG1 in sera from mice fed beans and peas and found that transgenic αAI and nGM peas produced high anti-pea lectin antibody titres that were higher than the other bean and pea seed meal fed-mice (Fig. 3B). These results indicated that the consumption of peas led to pea lectin antibody production. Secondly, we immunized mice i.p. with pea lectin and measured the anti-pea lectin IgG1 response (Fig. 3C) and also tested pea lectin immune sera against αAIs (Fig. 3D). As expected, immunization with pea lectin induced high serum titres when reacting against pea lectin. These anti-pea lectin antibodies also reacted against cowpea and pea αAIs and with less intensity to chickpea and bean αAIs. Taken together, these results demonstrate that feeding with transgenic and non-transgenic peas generates anti-pea lectin responses, which are cross-reactive with αAI and can be confused with anti-αAI antibodies.

To further evaluate immune responses generated by the consumption of pea and bean seed meals, we did an *in vivo* respiratory tract challenge with αAI to assess whether T cell priming occurred. To measure *in vivo* T cell immune responses, we instilled αAI into the nares of mice following 4 consecutive weeks of bean and pea feeding and measured leucocyte infiltration and mucus hypersecretion in lungs. Feeding beans and peas, whether raw or heat-treated, followed by i.n. αAI induced airway and lung inflammation, while gavage with PBS did not induce inflammation (Fig. 3E–G and Fig. S2). Similarly, all mice fed seed meal developed high levels of mucus secretion following i.n. αAI compared with PBS controls (Fig. 3H and Fig. S2).

Consumption of Pinto and Tendergreen bean seed meals led to the highest number of eosinophils in the airway with increased eosinophil recruitment in heat-treated compared to raw seed meal fed mice (Fig. 3E). In contrast, mice consuming raw transgenic peas had higher airway eosinophils compared to heat-treated peas. Tendergreen bean fed mice generated more extensive allergic lung inflammation than all the other seed meals (Fig. 3F and 3G). Both transgenic αAI and non-transgenic peas generated a severe inflammatory response in lung compared to Pinto bean, transgenic and nGM- cowpeas and chickpeas. We did not expect the responses to be higher in mice consuming heat-treated seed meals due to the denaturation of the proteins. However, we observed that some groups had higher eosinophilia in heat-treated compared to raw seed meals. We speculate that there are other components in the seeds that may affect the overall immune response to the seed meals and that these are influenced differentially during heat treatment.

**Figure 4:**
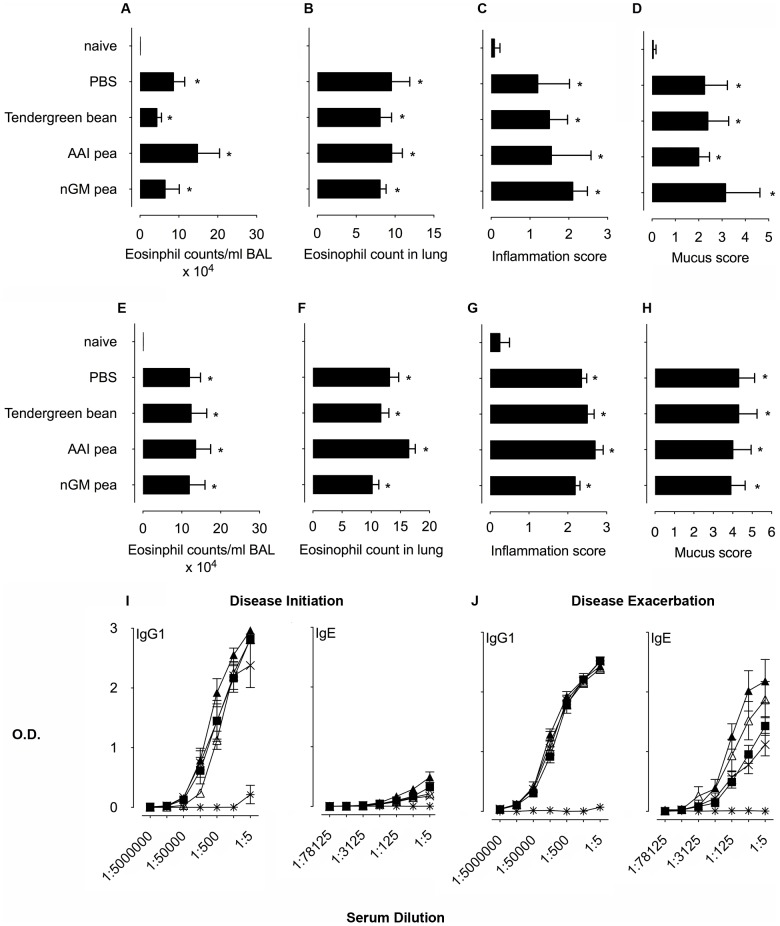
Adjuvant effect of αAI pea and bean consumption. Naïve BALB/c mice were compared with OVA-immunized and challenged mice gavaged with either PBS, or Tendergreen bean, αAI peas, non-transgenic pea seed meal. A. Eosinophil counts in BAL fluid from mice at disease initiation and e. exacerbation. B. Eosinophil counts in Luna-stained lung sections from mice at disease initiation and F. exacerbation. C. Inflammation scores of lung sections from mice at disease initiation and G. exacerbation. D. Mucus scores in PAS-stained lung sections from mice at disease initiation and H. exacerbation. Serum anti-OVA IgG1 and IgE antibody titres for mice at .I. disease initiation or J. disease exacerbation. Groups include naïve mice 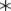, PBS alone ×, Tendergreen bean ▪, αAI pea ▴ and nGM pea ▵ gavaged mice. Data are expressed as mean OD_450 nm_ ± SEM; n = 8, duplicate samples. For IgE, dilutions are expressed ×10^3^. Data are expressed as means ± SEM; n = 8. For eosinophil counts in BAL and lungs, data were compared using the Kruskal-Wallis test followed by Dunn's multiple comparison test. For histological scoring, data were compared with the Mann Whitney test. *p<0.05 for all groups above the PBS controls. These are representative data from 2 experiments.

Although adjuvant studies are not routinely used in the assessment of GMOs, the effect of αAI peas on a non-crossreactive protein, OVA was previously tested and shown to enhance OVA-specific immunogenicity [8]. To test the effect of αAI pea feeding on immune responses to OVA, we used a different approach in models of OVA-induced allergic disease. We fed mice with seed meals during OVA sensitization and lung challenge for the onset of allergic disease (Fig. 1D) or fed mice before re-challenging with aerosolized OVA to induce disease exacerbation (Fig. 1E). OVA immunization and aerosol challenge generates an intense allergic response characterized by eosinophilic airway and lung inflammation, mucus hypersecretion and OVA-specific antibody responses [15]. After recuperation, chronic lung inflammatory infiltrates remain and respond to re-exposure to OVA leading to disease exacerbation for the lifetime of the mouse. To test the adjuvant effect of αAI peas, we gavaged mice twice weekly for 4 consecutive weeks with the transgenic αAI and nGM peas, Tendergreen beans or PBS before disease initiation and exacerbation. Naïve mice had healthy lungs and no αAI immune responses (Fig. 4). PBS control mice (OVA immunized and challenged, PBS gavaged), however, illustrate the response to OVA with approximately 30% and 40% eosinophils within the airways for disease initiation and exacerbation, respectively, while neither pea nor bean feeding influenced OVA-induced airway inflammation at either phase of disease (Fig. 4A and 4E). Consumption of peas and beans did not affect the OVA-specific eosinophilic inflammation, mucus secretion or severity of lung inflammation seen on Luna-, H&E- and PAS-stained tissue sections (Fig. 4B–H and Fig. S3 and S4). Antibody responses to OVA were unaffected by feeding αAI pea and bean (Fig. 4I and 4J).

In summary, our results show that there is variation in antibody responses to αAIs, but that there was not an increased antibody response to the αAIs from transgenic legumes compared to the αAIs from beans. αAIs from transgenic legumes and beans have minor differences in post-translational modifications that appear to modify immunogenicity [5]. However, we show here that these differences in immunogenicity did not differentiate αAIs from transgenic legumes with those found in beans. All αAIs induced high IgG1 antibody titres and are thus, immunogenic irrespective of transgenic or non-transgenic source. In feeding experiments, we observed that mice fed transgenic and non-transgenic legumes had immune and allergic responses that were similar to those generated by both Pinto and Tendergreen beans. Furthermore, the responses to the non-transgenic peas were related to a cross-reactive response to pea lectin and the consumption of transgenic, non-transgenic and bean seed meals did not accentuate allergic responses to another non-cross-reactive allergen.

Our results are at odds with the previous study in which mice developed allergic responses to αAI peas but not to beans [8], [16]. It is possible that the source of the mice and their normal baseline diets may play a role. The mice used in the Austrian experiments were purchased from Charles River Germany and maintained in a pathogen-free mouse room. The mice used in the Australian studies originated from the Jackson Laboratory and were bred at The John Curtin School of Medical Research by sibling mating for at least 70 generations in an SPF Unit. These mice were maintained in the Australian Phenomics Facility by inbred sibling mating. The health status of the mice in Austria revealed that there were no pathological or commensal organisms or antibodies detected. These data are not available for the mice used in Australia. There are no data regarding gut microbiota in either mouse house. The diet in Austria was from SSNIFF and the Australian diet was produced by Gordon's Specialty Stock Feeds P/L in New South Wales. The most obvious differences between the two diets are in the sources of the dietary protein (animal vs. plant), fatty acid type, level of soluble fibre and level of vitamin supplementation (Tables S1, S2, S3). While any or all of these dietary differences could influence immune responses, it is unlikely that they could cause a differential response to pea and bean constituents. Another possibility could be that αAI peas and proteins used in the studies differed, but the αAI peas and the non-transgenic controls were from the same batches of seeds produced at CSIRO. Because the previous study showed that only αAI peas caused allergic responses in mice, we were surprised that not only did Tendergreen bean and Pinto bean induce allergic responses, but so did the non-transgenic peas. We discovered that pea lectin antibodies are generated upon consumption of peas and that this antibody crossreacts with αAI.

In conclusion, although our studies show that consumption of both peas and beans leads to immune and allergic responses to αAI and pea lectin in mice, it is still not clear that these immune responses are biologically relevant for humans. In other words, it is not known whether these peas and beans would induce symptomatic allergic responses or indeed be relevant in human disease. These data derive from mice utilizing highly manipulative exposure regimens and therefore, do not provide definitive evidence that αAI peas would be allergenic in humans. Importantly, non-transgenic peas induced similar allergic responses compared to the transgenic peas. The reason for this response is related to cross-reactivity to another protein in peas. The response in this study to αAI in non-transgenic peas and beans is difficult to reconcile with the lack of response in Prescott et al. Moreover, bean allergies in patients are rare. This study emphasizes the importance of repeat experiments in independent laboratories and illustrates that unexpected cross-reactive allergic responses upon consumption of plant products can occur in mice. We recommend that the use of mouse models for testing GMO allergenicity needs to be carefully evaluated on a case-by-case basis.

## Supporting Information

Figure S1 Immune responses to αAIs upon i.n. immunization. Representative photomicrographs of lung from mice administered αAIs 6 times over a 3-week period. a. H&E stained lung sections at 10× objectives. b. PAS stained sections at 10× objective. These are representative data for individual mice (n = 8 in 2 experiments). Arrowheads indicate either areas of inflammation or mucus within lung epithelial goblet cells.
(TIF)

Figure S2 Inflammation and mucus secretion following consumption of raw αAI and nGM pea, chickpea and cowpea and Tendergreen and Pinto beans. Representative photomicrographs of lung from mice administered bean, transgenic and non-transgenic peas, chickpeas and cowpeas for 1 month. a. H&E stained lung sections at 10× objectives. b. PAS stained sections at 10× objective. These are representative data for individual mice (n = 8 in 2 experiments). Arrowheads indicate either areas of inflammation or mucus within lung epithelial goblet cells.
(TIF)

Figure S3 Adjuvant effect of consuming raw αAI pea and bean seed meals on acute disease initiation. Representative photomicrographs of lung from naïve BALB/c mice are compared with OVA-immunized and challenged mice gavaged with either PBS, or Tendergreen bean, αAI peas, nGM pea seed meal. a. H&E stained lung sections at 10× objectives. b. PAS stained sections at 10× objective. These are representative data for individual mice (n = 8 in 2 experiments). Arrowheads indicate either areas of inflammation or mucus within lung epithelial goblet cells.
(TIF)

Figure S4 Adjuvant effect of consuming αAI pea and bean seed meals on disease exacerbation. Representative photomicrographs of lung from naïve BALB/c mice are compared with OVA-immunized, challenged and then rechallenged mice gavaged with either PBS or Tendergreen bean, αAI peas, nGM pea seed meals. a. H&E stained lung sections at 10× objectives. b. PAS stained sections at 10× objective. Arrowheads indicate either areas of inflammation or mucus within lung epithelial goblet cells.
(TIF)

Table S1 Comparison of ingredients between Australian and Austrian diets.
(DOCX)

Table S2 Comparison of crude materials between Australian and Austrian diets.
(DOCX)

Table S3 Nutrient analysis Australian and Austrian diets.
(DOCX)
