# Supplementary figures and images for: Genetically Modified α-Amylase Inhibitor Peas Are Not Specifically Allergenic in Mice
Source: PLoS One. 2013 Jan 9;8(1):e52972. doi: 10.1371/journal.pone.0052972 (PMC3541390; doi:10.1371/journal.pone.0052972)

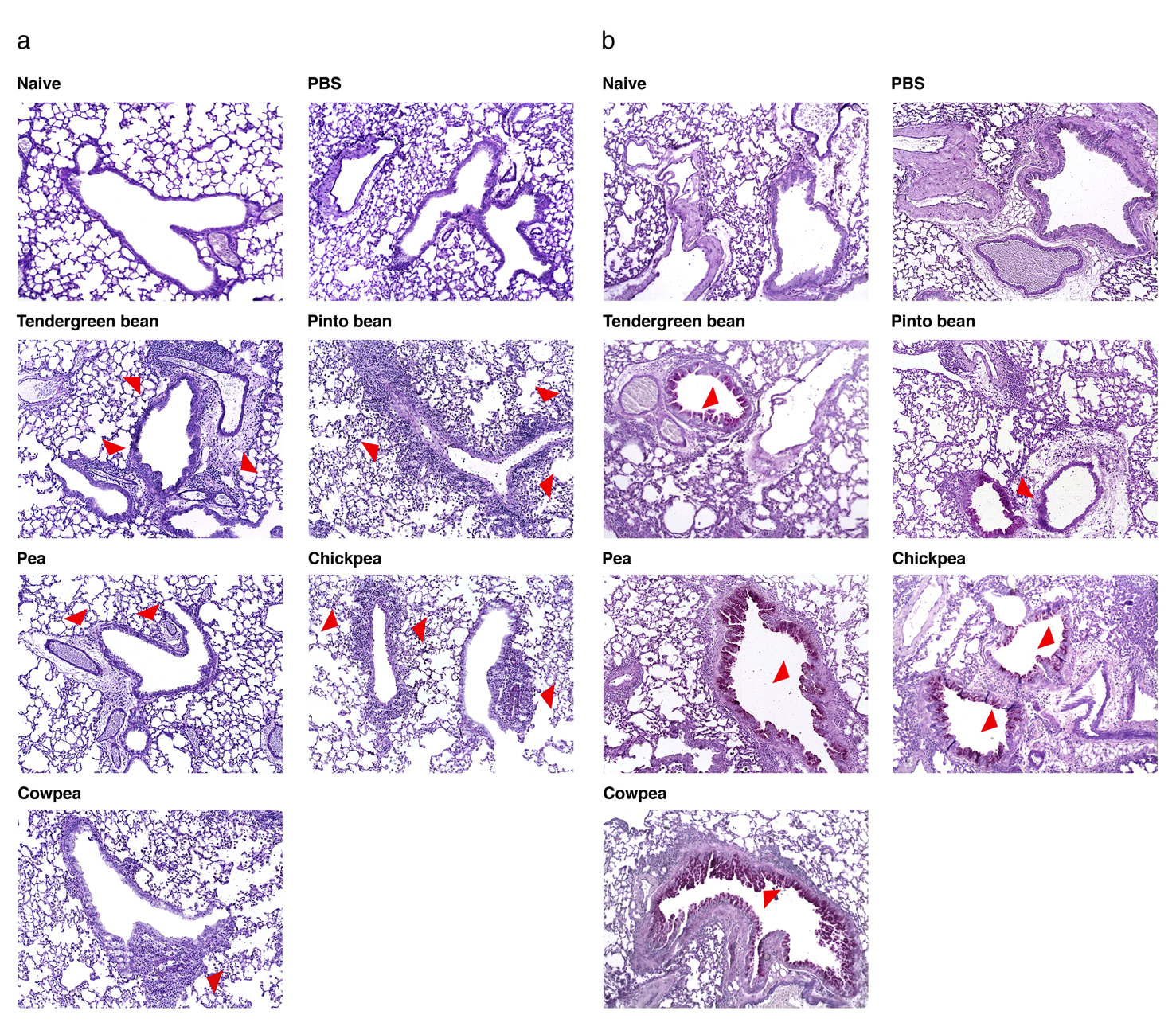

Supplement: Figure S1 — Immune responses to αAIs upon i.n. immunization. Representative photomicrographs of lung from mice administered αAIs 6 times over a 3-week period. a. H&E stained lung sections at 10× objectives. b. PAS stained sections at 10× objective. These are representative data for individual mice (n = 8 in 2 experiments). Arrowheads indicate either areas of inflammation or mucus within lung epithelial goblet cells. (TIF) [file pone.0052972.s001.tif]

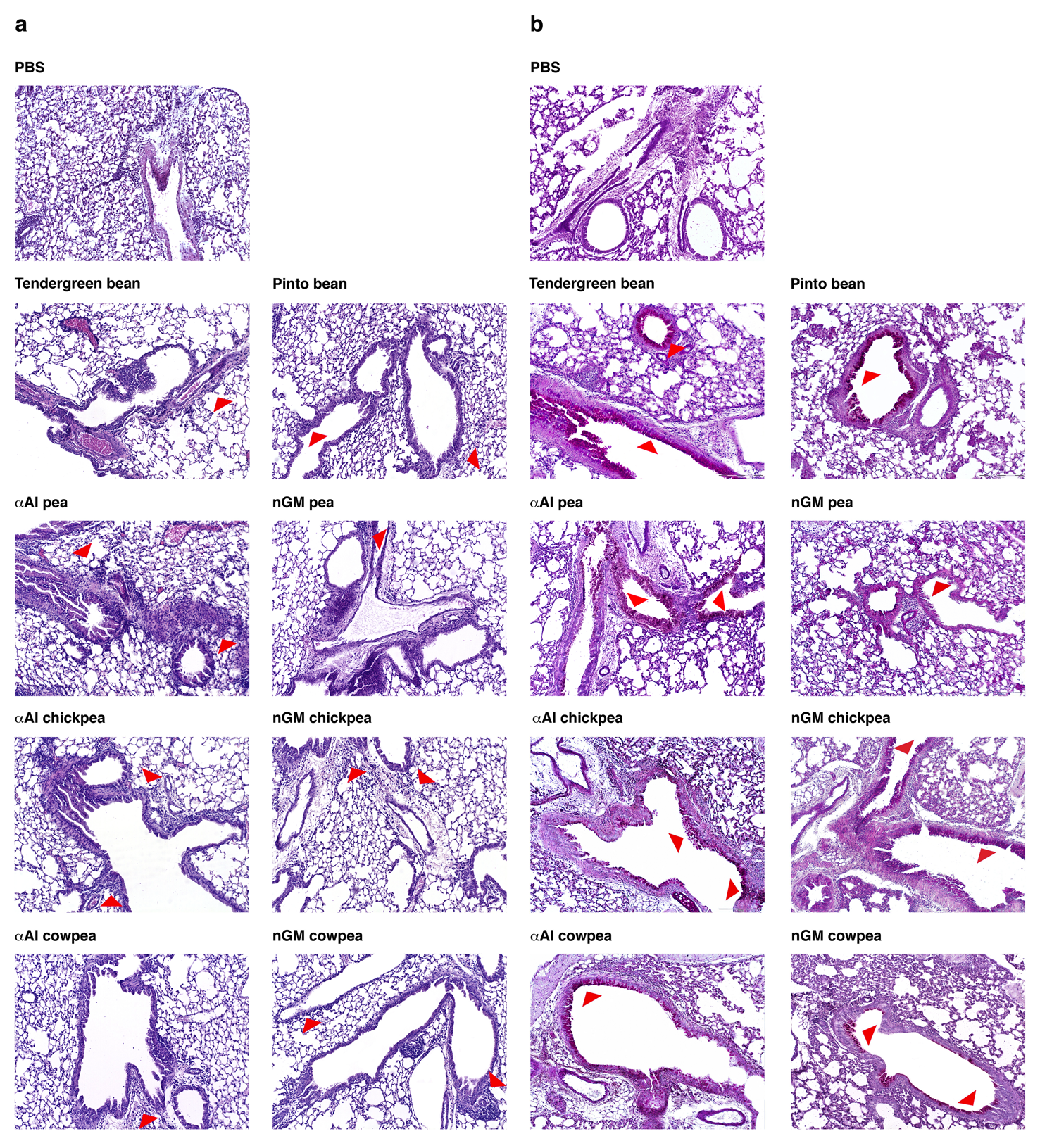

Supplement: Figure S2 — Inflammation and mucus secretion following consumption of raw αAI and nGM pea, chickpea and cowpea and Tendergreen and Pinto beans. Representative photomicrographs of lung from mice administered bean, transgenic and non-transgenic peas, chickpeas and cowpeas for 1 month. a. H&E stained lung sections at 10× objectives. b. PAS stained sections at 10× objective. These are representative data for individual mice (n = 8 in 2 experiments). Arrowheads indicate either areas of inflammation or mucus within lung epithelial goblet cells. (TIF) [file pone.0052972.s002.tif]

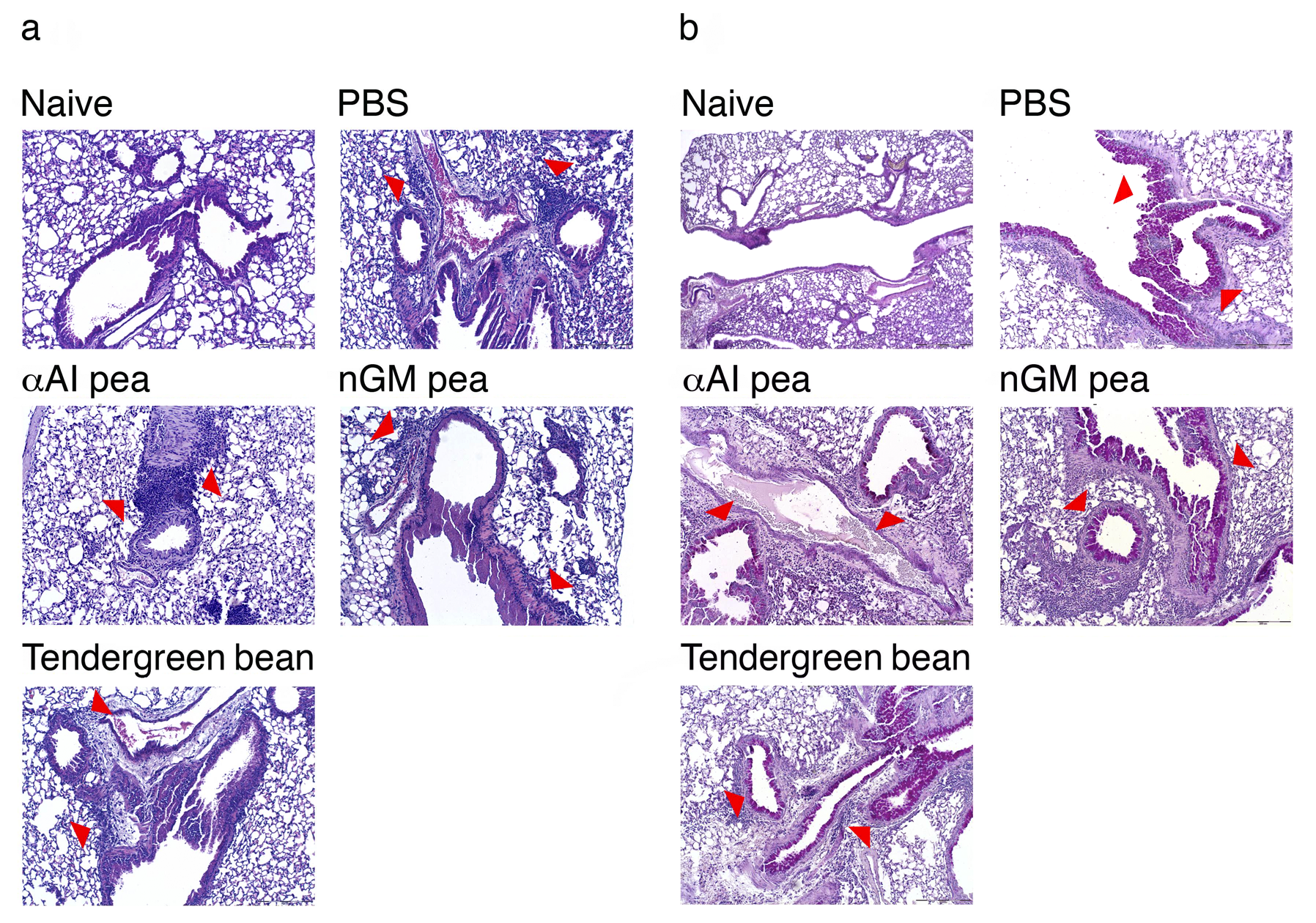

Supplement: Figure S3 — Adjuvant effect of consuming raw αAI pea and bean seed meals on acute disease initiation. Representative photomicrographs of lung from naïve BALB/c mice are compared with OVA-immunized and challenged mice gavaged with either PBS, or Tendergreen bean, αAI peas, nGM pea seed meal. a. H&E stained lung sections at 10× objectives. b. PAS stained sections at 10× objective. These are representative data for individual mice (n = 8 in 2 experiments). Arrowheads indicate either areas of inflammation or mucus within lung epithelial goblet cells. (TIF) [file pone.0052972.s003.tif]

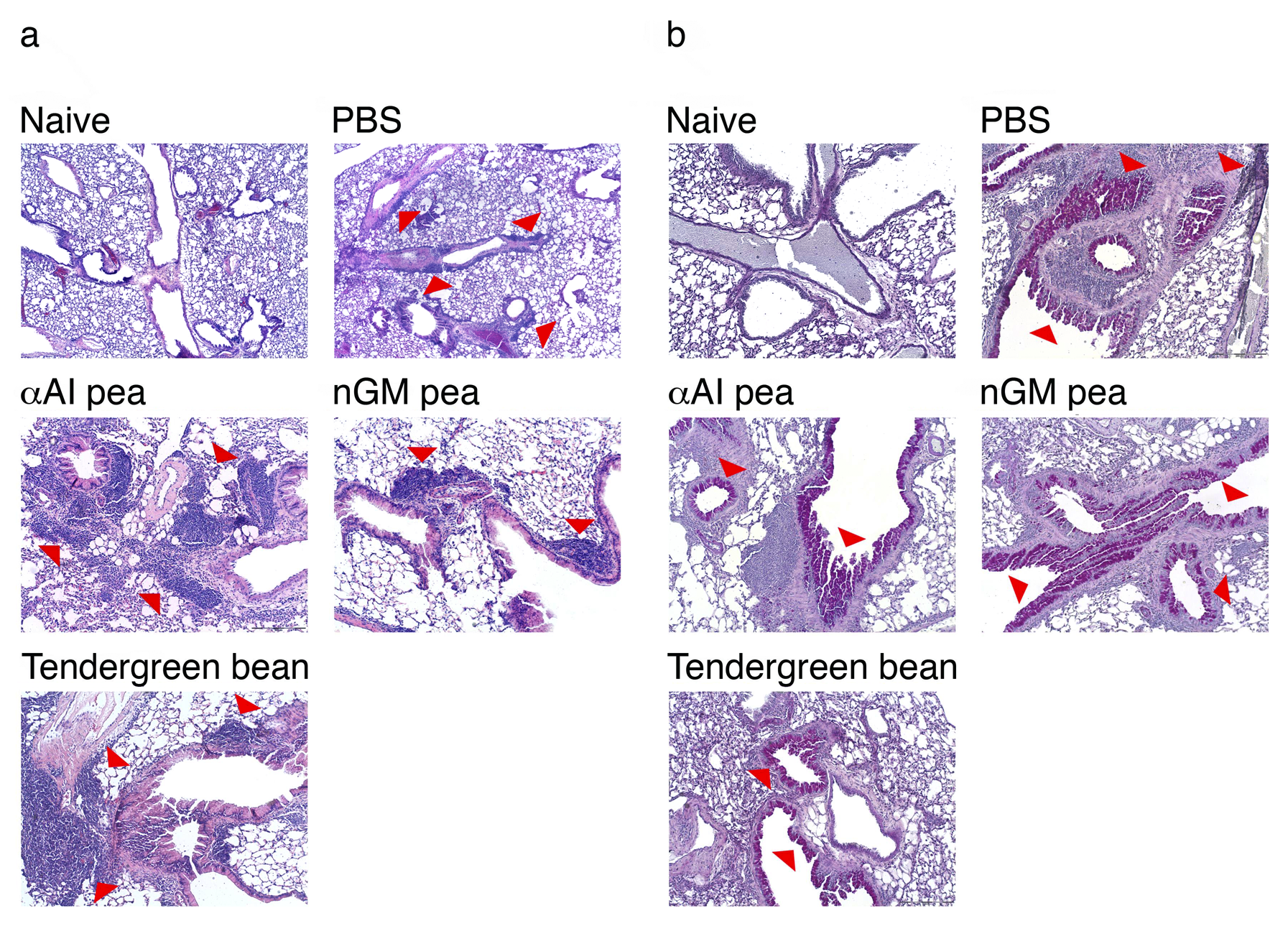

Supplement: Figure S4 — Adjuvant effect of consuming αAI pea and bean seed meals on disease exacerbation. Representative photomicrographs of lung from naïve BALB/c mice are compared with OVA-immunized, challenged and then rechallenged mice gavaged with either PBS or Tendergreen bean, αAI peas, nGM pea seed meals. a. H&E stained lung sections at 10× objectives. b. PAS stained sections at 10× objective. Arrowheads indicate either areas of inflammation or mucus within lung epithelial goblet cells. (TIF) [file pone.0052972.s004.tif]
