# Supplementary material for: Genetically Modified α-Amylase Inhibitor Peas Are Not Specifically Allergenic in Mice
Source: PLoS One. 2013 Jan 9;8(1):e52972. doi: 10.1371/journal.pone.0052972 (PMC3541390; doi:10.1371/journal.pone.0052972)
Supplement: Table S1 — Comparison of ingredients between Australian and Austrian diets. (DOCX) [file pone.0052972.s005.docx]

**Table S1: Comparison of ingredients between Australian and Austrian diets**

| Australian Diet* | Austrian diet** |
| --- | --- |
| Wheat | Wheat and wheat flour |
| Sorghum |  |
| Soybean meal | Soybean meal (full fat) |
| Pollard |  |
| Bran (wheat) | Bran (wheat) |
| Meat and bone meal |  |
| Blood meal |  |
| Fish meal |  |
| Lucerne meal |  |
| Canola oil | Soybean oil |
| Sunflower meal | Sunflower meal |
| Salt |  |
| Vitamin and mineral premix | Vitamin and mineral |
| Lysine |  |
| Choline chloride |  |
|  | Barley |
|  | Corn and corn products |
|  | Sugar beet pulp |

*Australian diet was from Gordon’s Specialty Feeds and the Austrian diet was from SSNIFF Germany.
