# Supplementary material for: Genetically Modified α-Amylase Inhibitor Peas Are Not Specifically Allergenic in Mice
Source: PLoS One. 2013 Jan 9;8(1):e52972. doi: 10.1371/journal.pone.0052972 (PMC3541390; doi:10.1371/journal.pone.0052972)
Supplement: Table S2 — Comparison of crude materials between Australian and Austrian diets. (DOCX) [file pone.0052972.s006.docx]

**Table S2: Comparison of crude materials between Australian and Austrian diets**

|  | **Australian diet** | **Austrian diet** |
| --- | --- | --- |
| Min. Crude Protein | 23% | 22.1 % |
| Min. Crude Fat | 6% | 4.5 % |
| Max. Crude Fibre | 5% | 3.9 % |
| M.E. (Min.) | 13 MJ/kg | (estimated:14.5 MJ/kg) |
| Dry substance | (suggested: 90%) | 90.4 % |
| Crude Ash | (suggested: 7%) | 6.7 % |
| N-free extract materials | (estimated: 49%) | 53.3 % |
| Starch | 30.7% | 35.8 % |
| Sugar | 4.1% | 5.2 % |

*Australian diet was from Gordon’s Specialty Feeds and the Austrian diet was from SSNIFF Germany.
