# Supplementary material for: Genetically Modified α-Amylase Inhibitor Peas Are Not Specifically Allergenic in Mice
Source: PLoS One. 2013 Jan 9;8(1):e52972. doi: 10.1371/journal.pone.0052972 (PMC3541390; doi:10.1371/journal.pone.0052972)
Supplement: Table S3 — Nutrient analysis Australian and Austrian diets. (DOCX) [file pone.0052972.s007.docx]

**Table S3: Nutrient analysis Australian and Austrian diets**

| **Amino Acids** | **g/kg** | **% (g/kg)** |
| --- | --- | --- |
| Lysine | 9.8 | 1.41 (14.1) |
| Methionine & Cystine | 5.9 | 0.83 (8.3) |
| Threonine | 8.4 | 0.76 (7.6) |
| Histidine | 5.0 | 0.51 (5.1) |
| Leucine | 15.2 | 1.5 (15) |
| Arginine | 12.1 | 1.31 (13.1) |
| Valine | 10.2 | 1.00 (10) |
| Isoleucine | 8.0 | 0.89 (8.9) |
| Phenylaline & Tyrosine | 16.4 | 1.64 (16.4) |
| Tryptophan | 3.7 | 0.28 (2.8) |
| Methionine |  | 0.46 (4.6) |
| Leucine | 17.3 | 1.5 (15) |
| Phenylalanine |  | 0.97 (9.7) |
| Glycin |  | 0.91 (9.1) |
| **Minerals** |  |  |
| Calcium | 10.1 g/kg | 1% (10 g/kg) |
| Phosphorus | 7.7 g/kg | 0.7% (7 g/kg) |
| Potassium | 5.4 g/kg | 1% (10 g/kg) |
| Magnesium | 1.8 g/kg | 0.24% (2.4 g/kg) |
| Iron | 97.0 mg/kg | 174 mg/kg |
| Copper | 10.6 mg/kg | 16 mg/kg |
| Manganese | 87.4 mg/kg | 73 mg/kg |
| Zinc | 48.1 mg/kg | 98 mg/kg |
| Iodine | 1.15 mg/kg | 2.2 mg/kg |
| Selenium | 0.1 mg/kg | 0.3 mg/kg |
| Sodium | 0.3% | 0.25% |
| Cobalt | - | 2.2 mg/kg |
| **Fats** | **% of lipid** | **% of lipid** |
| Saturated Fat | 21.3 | 16.4 |
| Mono-unsaturated | 42.9 | 22.4 |
| Poly-unsaturated | 30.7 | 61.2 |
| **Fatty acids** |  | **% of diet** |
| C 14:0 | 1.1 | 0.01 |
| C 16:0 | 13.6 | 0.56 |
| C 16:1 palmitel acid | <0.1 | 0.02 |
| C 18:0 | 4.5 | 0.14 |
| C 18:1 elaidic acid | <0.1 | 0.96 |
| C 18:2 linoleic acid | -26.4 | 2.42 |
| C 18:3 | 5.2 | 0.31 |
| C 20:0 | 0.4 | 0.02 |
| C 20:1 | 0.8 | 0.02 |
| C 20:5 | - | - |
| C 22:6 | - | - |
| **Vitamins** | **g/kg** | **per kg** |
| Vitamin A | 170 µg/100g | 25.000 IE (750 ug/100g) |
| Vitamin B1 | 4 mg/kg | 87 mg |
| Vitamin B2 | 5 mg/kg | 32 mg |
| Vitamin B6 | 6 mg/kg | 32 mg |
| Vitamin B12 | 0.005 mg/kg | 150 µg (0.150 mg/kg) |
| Vitamin C | 150 mg/kg | - |
| Vitamin D | 200 i.u/kg | 1000 IE |
| Vitamin E | 50 mg/kg | 138 mg |
| Vitamin K | 5 mg/kg | 20 mg |
| Niacin | 10 mg/kg | 165 mg |
| Pantothenate | 12 mg/kg | 62 mg |
| Folic Acid | 10 mg/kg | 10 mg |
| Biotin | 0.06 mg/kg | 730 µg (0.73 mg/kg) |
| Cholin-Cl |  | 3.300 mg |
| Inositol |  | 100 mg |

*Australian diet was from Gordon’s Specialty Feeds and the Austrian diet was from SSNIFF Germany.
